# Selective HDAC inhibition by ACY-241 enhances the activity of paclitaxel in solid tumor models

**DOI:** 10.18632/oncotarget.13738

**Published:** 2016-12-01

**Authors:** Pengyu Huang, Ingrid Almeciga-Pinto, Matthew Jarpe, John H van Duzer, Ralph Mazitschek, Min Yang, Simon S Jones, Steven N Quayle

**Affiliations:** ^1^ Acetylon Pharmaceuticals, Inc., Boston, MA 02210, USA; ^2^ Center for Systems Biology, Massachusetts General Hospital, Harvard Medical School, Boston, MA, USA

**Keywords:** HDAC, paclitaxel, solid tumors, mitotic spindle

## Abstract

ACY-241 is a novel, orally available and selective histone deacetylase (HDAC) 6 inhibitor in Phase 1b clinical development in multiple myeloma (NCT 02400242). Like the structurally related drug ACY-1215 (ricolinostat), ACY-241 has the potential for a substantially reduced side effect profile versus current nonselective HDAC inhibitor drug candidates due to reduced potency against Class I HDACs while retaining the potential for anticancer effectiveness. We now show that combination treatment of xenograft models with paclitaxel and either ricolinostat or ACY-241 significantly suppresses solid tumor growth. In cell lines from multiple solid tumor lineages, combination treatment with ACY-241 and paclitaxel enhanced inhibition of proliferation and increased cell death relative to either single agent alone. Combination treatment with ACY-241 and paclitaxel also resulted in more frequent occurrence of mitotic cells with abnormal multipolar spindles and aberrant mitoses, consistent with the observed increase of aneuploid cells. At the molecular level, multipolar mitotic spindle formation was observed to be NuMA-dependent and γ-tubulin independent, suggesting that treatment-induced multipolar spindle formation does not depend on centrosomal amplification. The significantly enhanced efficacy of ACY-241 plus paclitaxel observed here, in addition to the anticipated superior safety profile of a selective HDAC6 inhibitor versus pan-HDAC inhibitors, provides a strong rationale for clinical development of this combination in patients with advanced solid tumors.

## INTRODUCTION

Paclitaxel is a chemotherapeutic agent approved for use in the treatment of multiple solid tumor types, including breast cancer, ovarian cancer, non-small cell lung cancer and Kaposi's sarcoma [1–3]. As a microtubule-stabilizing drug, high concentrations of paclitaxel induce mitotic arrest followed by apoptosis or mitotic slippage, which had been recognized as the major mechanism of action for the treatment of tumors [4–7]. However, recent studies indicate that lower concentrations of paclitaxel, similar to levels observed within the tumors of treated patients, caused multipolar mitotic spindle formation, subsequently resulting in aberrant mitosis and cell death, which likely also contributes to the anti-cancer efficacy of paclitaxel [8, 9]. Consistent with this discovery, live cell imaging showed that paclitaxel treatment suppressed microtubule dynamic instability and induced multi-aster formation [10].

Dynamic instability is a critical regulator of microtubule biology. Microtubules are complex polymers that repeatedly undergo rapid and stochastic transitions between growth and contraction, thus enabling localized changes for specific physiologic purposes, for example, mitotic spindle formation during mitosis [11, 12]. This instability is tightly regulated by multiple posttranslational modifications, including acetylation of lysine-40 of α-tubulin [13, 14], but is also sensitive to microtubule disrupting agents, including paclitaxel and other taxane drugs [15, 16]. Amongst many known regulators of α-tubulin posttranslational modification, histone deacetylase (HDAC) 6 is the major deacetylase of lysine-40 [17].

HDAC inhibitors are an emerging class of anticancer agents that includes the FDA approved drugs vorinostat, romidepsin, and belinostat for the treatment of cutaneous and/or peripheral T cell lymphoma, and panobinostat for the treatment of multiple myeloma. Non-selective pan-HDAC inhibitors, that broadly target multiple members of the HDAC enzyme family, result in anticancer efficacy through regulation of the acetylation state of lysine residues in both histone and non-histone proteins [18–22]. Combinations of HDAC inhibitors with chemotherapeutic agents, including taxanes, have demonstrated evidence of clinical benefit, for example in ovarian cancer combining belinostat with carboplatin and paclitaxel [23], and in advanced non-small-cell lung cancer (NSCLC) combining vorinostat with carboplatin and paclitaxel [24]. Preclinical enhancement of combination efficacy of HDAC inhibitors and paclitaxel has also been demonstrated in models of ovarian, anaplastic thyroid, and breast cancer [25–27]. Though the underlying mechanism is still not fully understood, these studies did demonstrate enhancement of antitumor activity in combination with multiple pan-selective HDAC inhibitors, including vorinostat, valproic acid, and romidepsin. Unfortunately, the toxicity profile of pan-HDAC inhibitors frequently limits their use in combination with other drugs commonly used in the treatment of hematologic and solid tumors [19, 20].

Such tolerability concerns have motivated the identification and development of more selective HDAC inhibitors with improved safety profiles. Ricolinostat, the first-in-class HDAC6 selective inhibitor, has recently demonstrated acceptable tolerability and preliminary evidence of anti-myeloma efficacy upon combination treatment with lenalidomide and dexamethasone, as well as pharmacodynamic evidence of both HDAC6 and Class I HDAC inhibition in patients [28]. Amongst other effects, inhibition of HDAC6 results in hyperacetylation of α-tubulin, which has been shown to increase microtubule stability against cold and nocodazole induced depolymerization [13], suggesting that inhibition of HDAC6 by selective or pan-HDAC inhibitors may contribute to enhanced efficacy in combination with taxanes.

Here, paclitaxel treatment in combination with ACY-241, a second generation HDAC6 selective drug, further impacted the growth and viability of cell lines derived from multiple solid tumor lineages. Combination treatment caused significantly increased inhibition of tumor cell proliferation through enhanced reduction of S-phase, and was associated with increased frequency of abnormal multipolar mitotic spindle formation, induction of aneuploidy, and increased cell death. These findings provide supportive rationale for the clinical evaluation of ACY-241 in combination with paclitaxel in patients with advanced solid tumors, which is currently being tested in clinical trial NCT02551185.

## RESULTS

### Selective inhibition of HDAC6 by ACY-241

**Figure 1:**
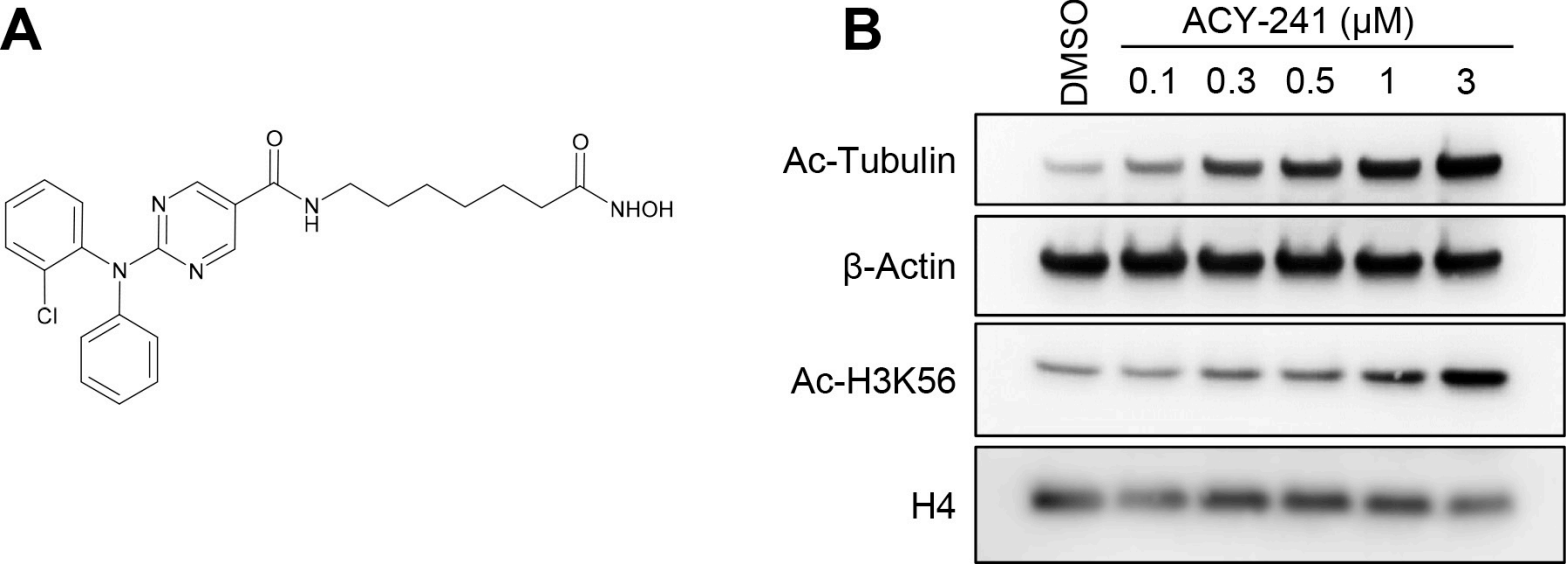
ACY-241 selectively inhibits HDAC6 (**A**) Chemical structure of ACY-241. (**B**) A2780 cells were cultured with vehicle or a range of ACY-241 concentrations for 24 hours prior to immunoblotting. ACY-241 preferentially induced hyperacetylation of α-tubulin relative to H3K56.

ACY-241 was designed as a second generation orally available and HDAC6 selective inhibitor with improved solubility properties over the structurally related inhibitor ricolinostat (Figure 1A; Table 1). Like ricolinostat, ACY-241 exhibits potent biochemical inhibition of HDAC6 with 13–18-fold reduced potency against the nuclear Class I HDACs (HDAC1, HDAC2, and HDAC3; Table 1). In A2780 ovarian cancer cells, 24 hour treatment with 300 nM ACY-241 resulted in increased hyperacetylation of α-tubulin, consistent with inhibition of the tubulin deacetylase HDAC6 (Figure 1B). In contrast, hyperacetylation of histone H3, a target of Class I HDACs, was only observed at doses above 1 μM. This result confirms that low exposures of ACY-241 result in selective inhibition of HDAC6, while higher exposures leads to inhibition of Class I HDAC isozymes.

**Table 1:** Inhibition of HDAC enzymes by ACY-241

Enzyme	IC_50_ (nM)	Potency (fold) vs HDAC6
**HDAC1**	35	13
**HDAC2**	45	17
**HDAC3**	46	18
**HDAC4**	> 20 000	> 7600
**HDAC5**	> 20 000	> 7600
**HDAC6**	2.6	–
**HDAC7**	7 300	2800
**HDAC8**	137	53
**HDAC9**	> 20 000	> 7600

### Combination treatment with paclitaxel reduced xenograft growth and cellular proliferation of solid tumor cancer cells

**Figure 2:**
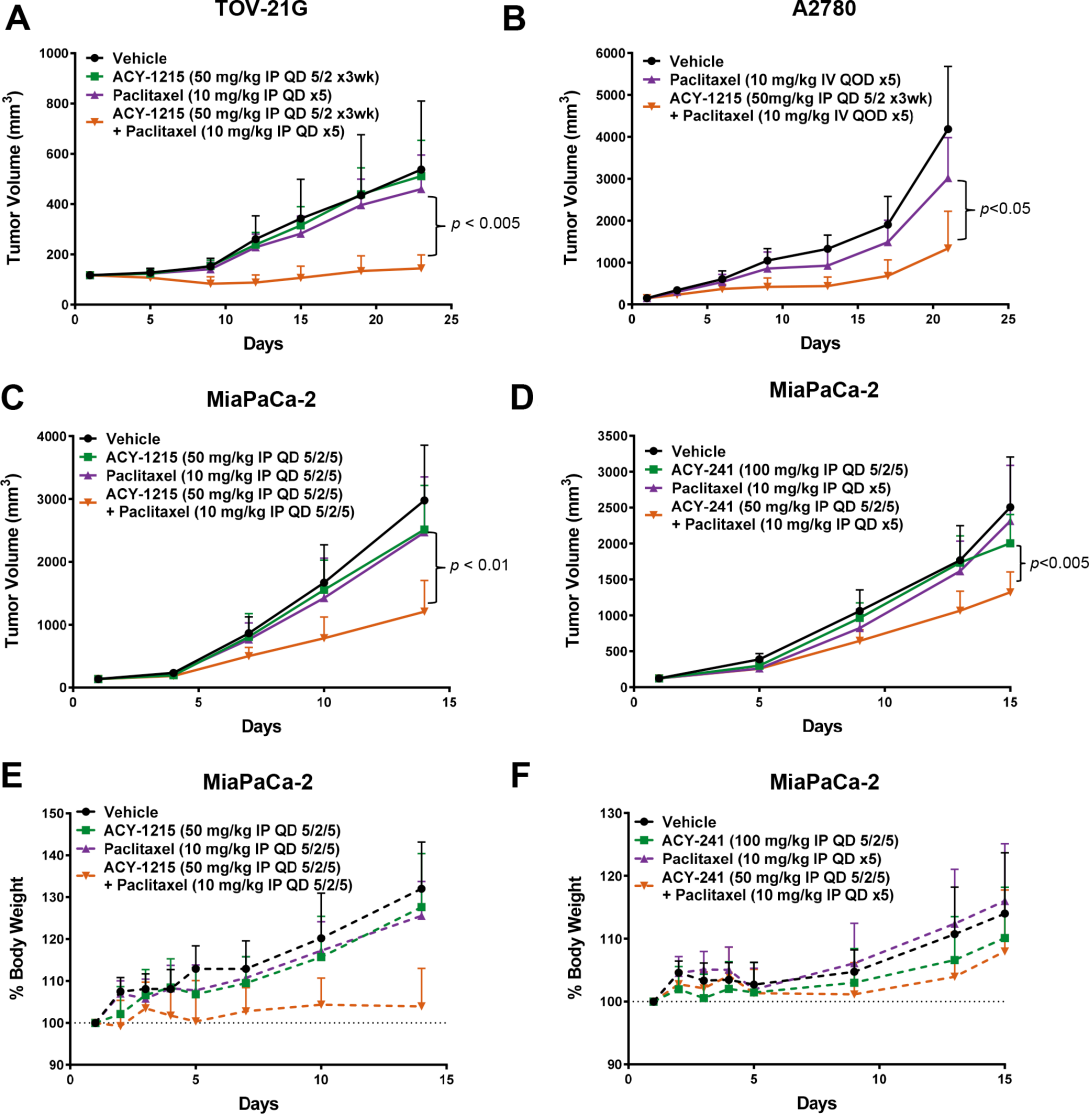
Combination treatment with HDAC6 inhibitors and paclitaxel reduced tumor xenograft growth Combination treatment of (**A**) TOV-21G and (**B**) A2780 ovarian cancer with ricolinostat plus paclitaxel resulted in significantly greater suppression of tumor growth relative to control and single agent treatments. Combination treatment of MiaPaCa-2 pancreatic cancer xenografts with ricolinostat (**C**) or ACY-241 (**D**) plus paclitaxel resulted in similar suppression of tumor growth. Shown is the mean tumor volume ± SD over the course of the treatment period. (**E**, **F**) Combination treatment of the MiaPaCa-2 model with ACY-1215 or ACY-241 did not result in body weight loss. Shown is the mean relative body weight change ± SD over the course of the treatment period.

Based on prior evidence of efficacy from paclitaxel and HDAC inhibitor combination treatment, the anti-cancer potential of paclitaxel in combination with selective HDAC6 inhibitors was assessed in multiple solid tumor xenograft models, including those of ovarian and pancreatic cancer origins. Combination treatment of TOV-21G, A2780, and MiaPaCa-2 xenografts with ricolinostat plus paclitaxel resulted in significantly greater suppression of tumor growth relative to either single agent (Figure 2A and 2C, respectively). Likewise, ACY-241 exhibited similarly significant efficacy in combination with paclitaxel in the MiaPaCa-2 xenograft model (Figure 2D). Importantly, ricolinostat and ACY-241 were well tolerated in mice when dosed as a single agent and in combination with paclitaxel (Figure 2E and 2F, respectively). Together, these results demonstrate substantial anti-cancer efficacy of both ACY-241 and ACY-1215 in combination with paclitaxel in xenograft models.

The potential of ACY-241 and paclitaxel to reduce proliferation upon combination treatment was also assessed in cell lines generated from multiple solid tumor types (e.g. pancreatic, ovarian, and breast cancer). The single agent viability IC_50_ of ACY-241 and paclitaxel ranged from 4.6 – 6.1 μM and 2.3 – 3.2 nM, respectively, in A2780 and TOV-21G ovarian cancer and MDA-MD-231 breast cancer cells (Supplementary Figure S1A, S1B). Single agent dose response was also assessed in a long term live-cell imaging proliferation assay using the IncuCyte^®^ platform (Supplementary Figure S1C, S1D). Briefly, MDA-MB-231 breast cancer cells stably expressing nucleus-restricted Red Fluorescent Protein were incubated with the indicated doses of each compound alone or in combination and absolute cell number was determined in real-time (Supplementary Figure S1C). Additionally, non-toxic Caspase 3/7 Reagent was added to each culture well for real-time detection of the induction of apoptosis due to substrate cleavage by Caspase 3/7 (Supplementary Figure S1D). Consistent with the viability assay, single agent ACY-241 modestly reduced proliferation at doses up to 3 μM without inducing apoptosis, while 10 μM of ACY-241 caused significant induction of apoptosis and completely suppressed proliferation (Supplementary Figure S1C, S1D; *left panels*). Similarly, single agent paclitaxel modestly reduced cell proliferation up to 2 nM and did induce apoptosis after longer duration of exposure (Supplementary Figure S1C, S1D; *middle panels*).

**Figure 3:**
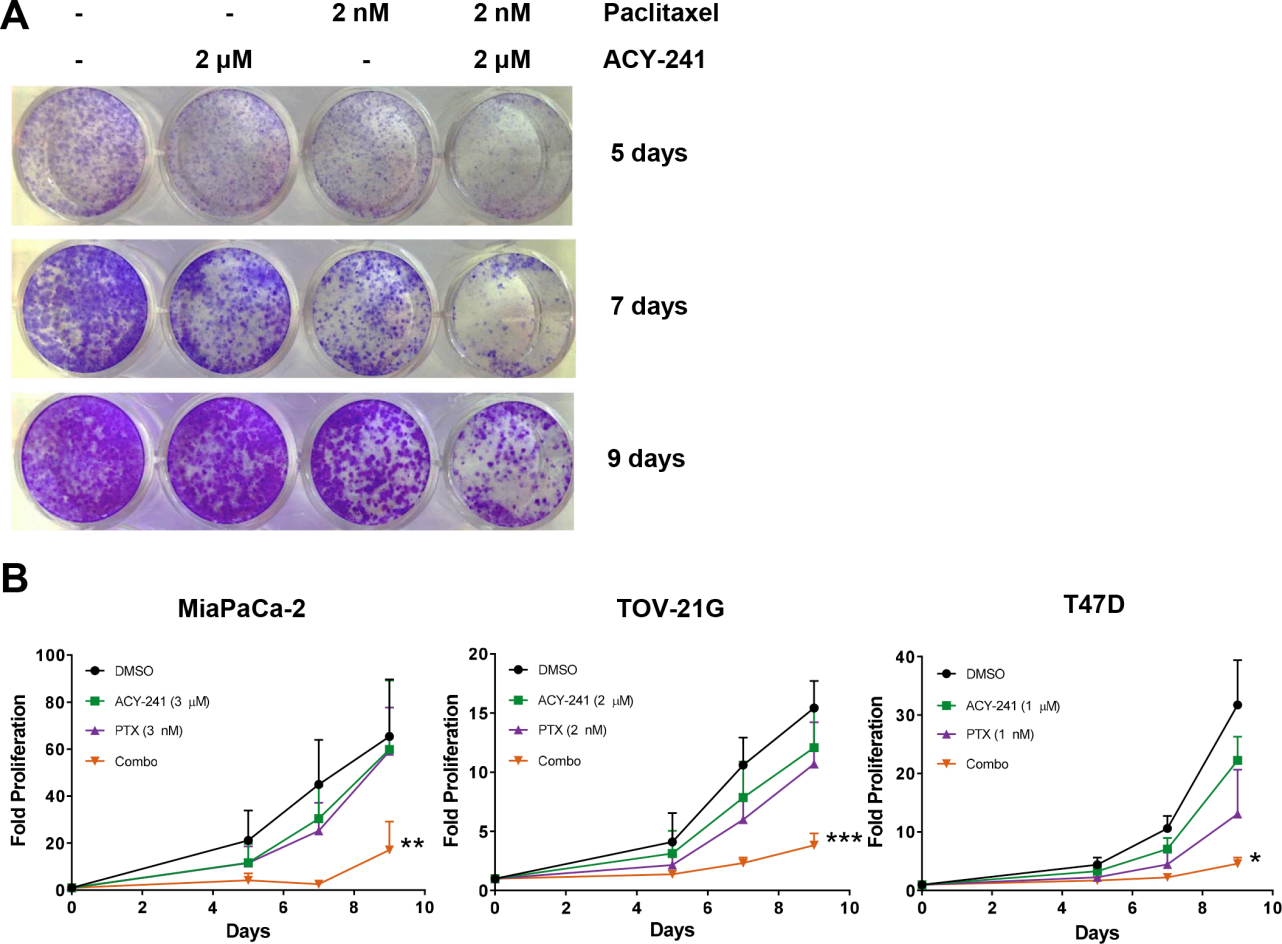
Combination treatment with ACY-241 and paclitaxel reduced cancer cell proliferation MiaPaCa-2, TOV-21G, or T47D cells were incubated with the indicated concentrations of ACY-241 and/or paclitaxel for 9 days. (**A**) Representative image of TOV-21G cell proliferation in response to treatment with each agent as assessed by crystal violet staining. (**B**) Relative proliferation was plotted as mean total crystal violet absorption of three independent experiments ± SD over time. *p < 0.05, **p < 0.005, and ***p < 0.0001

*In vitro* combination efficacy was initially assessed in the MDA-MB-231 cell line by selecting single agent doses of each agent that only partially suppressed proliferation. Upon combination treatment there was significantly reduced proliferation and enhanced apoptosis relative to either single agent (Supplementary Figure S1C, S1D; *right panels*). To further assess combination efficacy, MiaPaCa-2 pancreatic cancer, TOV-21G ovarian cancer, and T47D breast cancer cells were treated with ACY-241, paclitaxel, or the combination and relative cell proliferation was monitored over a 9 day treatment period (Figure 3A, 3B). While each single agent was only partially active at the selected concentrations, combination treatment with ACY-241 and paclitaxel significantly inhibited proliferation and this effect was maintained throughout the treatment period (Figure 3B).

### Enhanced induction of cell cycle arrest and cell death by ACY-241 in combination with paclitaxel

**Figure 4:**
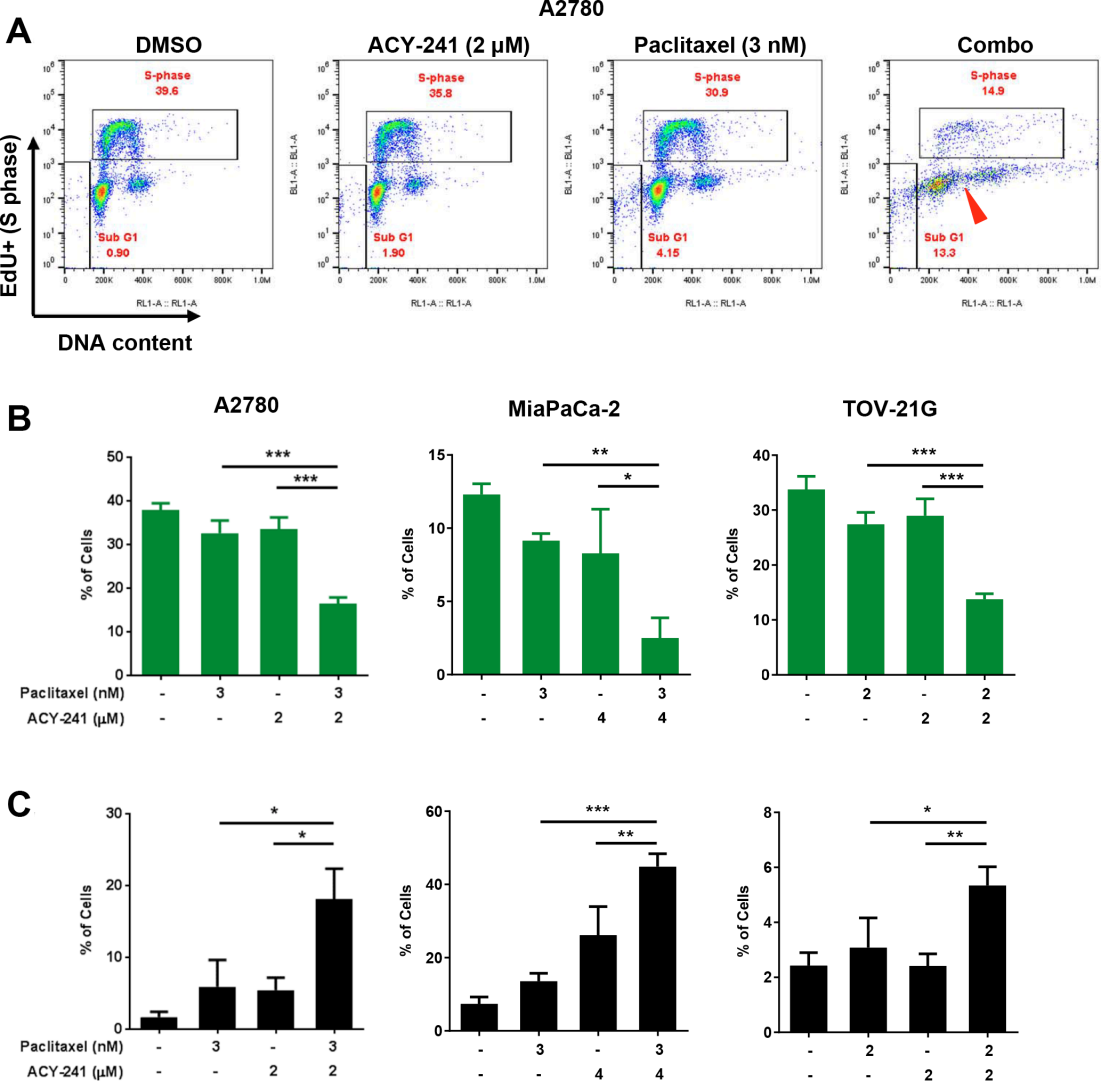
Reduction of S-phase population, induction of cell death and impact on DNA content after combination treatment with ACY-241 and paclitaxel (**A**) Dual parameter Click-iT Plus Edu (y axis, DNA synthesis) and FxCycle Far Red (x axis, DNA content) staining in A2780 ovarian cancer cell cells indicate the percentage of cells in S-phase and SubG1 after 3 days of treatment with ACY-241, paclitaxel, or the combination. In addition to reduced S-phase and increased subG1 frequency, combination treatment results in an aneuploid cell population (DNA content distributed between 2 N and 4 N; red arrowhead). Percentage of cells in S-phase (**B**) or in the SubG1 population (**C**) was determined after treatment of A2780 or TOV-21G ovarian cancer (3 days) or MiaPaCa-2 pancreatic cancer cells (5 days) with the indicated concentrations of each agent. Shown is the mean ± SD of three independent experiments. *p < 0.05, **p < 0.005, and ***p < 0.0001

To assess if decreased proliferation after combination treatment was associated with effects on cell cycle progression, A2780 ovarian cancer cells were treated with ACY-241 (2 μM), paclitaxel (3 nM), or the combination of both agents, and relative cell cycle distribution was assessed by EdU incorporation. As shown in the flow cytometry dot plots in Figure 4A, vehicle treated cells were actively replicating DNA in S phase, and populations of cells with distinct DNA content (representing G1 and G2/M phase cells) were also detected. Similar populations were observed after treatment with either ACY-241 or paclitaxel as single agents, but after combination treatment fewer S phase cells were detected and the remaining cell populations exhibited an irregular distribution of DNA content (Figure 4A). This observation suggests that combination treatment results in reduced cell cycle progression and accumulation of cells with variable DNA content, consistent with the induction of aneuploidy due to a possible defect in mitosis.

Analysis of multiple cell lines (A2780 and TOV-21G ovarian cells; MiaPaCa-2 pancreatic cancer cells) confirmed that combination treatment with ACY-241 and paclitaxel significantly reduced the percentage of cells in S phase, thus reducing cell cycling (Figure 4B). The reduction in S phase cells after combination treatment was significantly enhanced over either single agent at these concentrations. Consistent with prior studies [8, 9], high concentrations of paclitaxel (100 nM) alone induced cell cycle arrest at mitosis (M) phase, as demonstrated by increasing frequency of phospho-histone H3 (pH3) positive cells from 8 to 24 hours after treatment (Supplementary Figure S2A). In contrast, low dose paclitaxel (6 nM) resulted in accumulation of M phase cells (14% pH3 positive cells vs 2.6% pH3 positive with DMSO treatment) within 8 hours of treatment, consistent with the induction of spindle assembly checkpoint (SAC)-dependent mitotic arrest at this dose level. However, most of the accumulated mitotic cells under low dose treatment eventually resolved their arrested mitoses with prolonged treatment time. This is indicated by the decreased percentage of pH3 positive cells (4.1% pH3 positive cells) after longer treatment time (24 hours), suggesting these cells overcame a cell cycle checkpoint and resolved mitosis. Similar results were also observed upon combination treatment with ACY-241 and paclitaxel, further supporting an apparent impact of the treatment on mitosis (Supplementary Figure S2A). Consistent with this observation, cell morphology also showed that cells treated with the combination of ACY-241 with low dose paclitaxel were able to exit mitosis and reattach to the culture surface, while cells treated with high dose paclitaxel (100 nM) were arrested at M phase and remained spherical (Supplementary Figure S2B). Thus, combination treatment at these doses significantly reduced cell cycling but did not completely block cell cycle progression. Beyond reduction of cell cycling, combination treatment also significantly increased induction of cell death relative to control or single agent treatment (Figure 4C). Consistent with reduced proliferation and inhibition of tumor growth, these findings confirm that ACY-241 treatment in combination with paclitaxel reduces cell cycle progression and results in the accumulation of cells with abnormal DNA content.

### Combination treatment with ACY-241 and paclitaxel enhances α-tubulin acetylation

**Figure 5:**
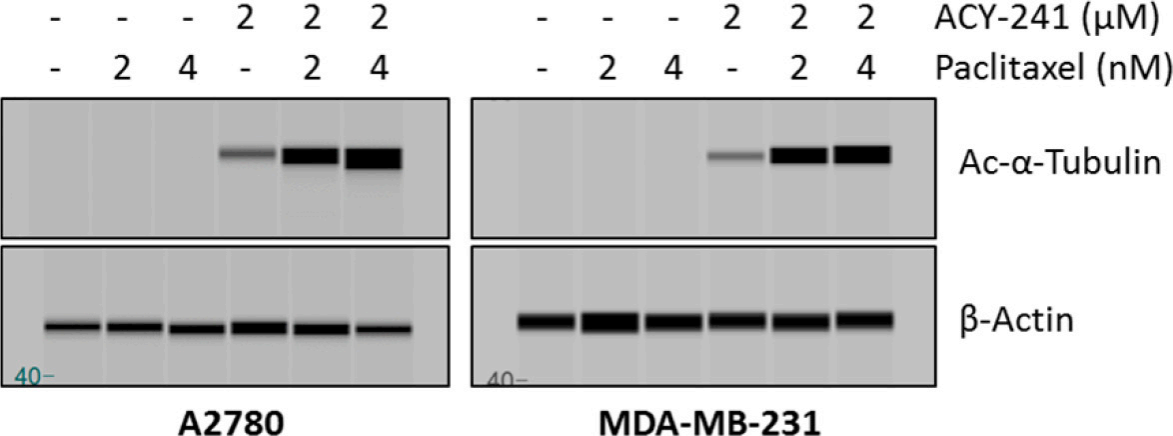
Combination treatment caused enhanced hyperacetylation of α-tubulin Immunoblotting demonstrated enhanced hyperacetylation of α-tubulin after combination treatment with ACY-241 and paclitaxel in A2780 ovarian cancer and MDA-MB-231 breast cancer cells. A2780 and MDA-MB-231 cells were treated for 48 hours with the indicated concentrations of each drug and whole cell protein lysates were subjected to immunoblotting using antibodies for acetylated-α-tubulin and β-actin.

At the molecular level, treatment with paclitaxel results in enhanced stability of microtubules, which in turn leads to increased acetylation of α-tubulin [29]. Likewise, HDAC6 actively regulates acetylation of α-tubulin, and ACY-241 treatment results in a dose-dependent increase in α-tubulin hyperacetylation (Figure 1B). Upon combination treatment with ACY-241 and paclitaxel a synergistic increase in α-tubulin hyperacetylation was detected in A2780 and MDA-MB-231 cells (Figure 5). Thus, the enhanced anti-cancer efficacy resulting from combination treatment with ACY-241 and paclitaxel in cell lines was associated with further increased hyperacetylation of α-tubulin, suggesting these agents synergistically impact the regulation of tubulin biology.

### Combination of ACY-241 and paclitaxel increases the frequency of abnormal multipolar spindle formation during mitosis

**Figure 6:**
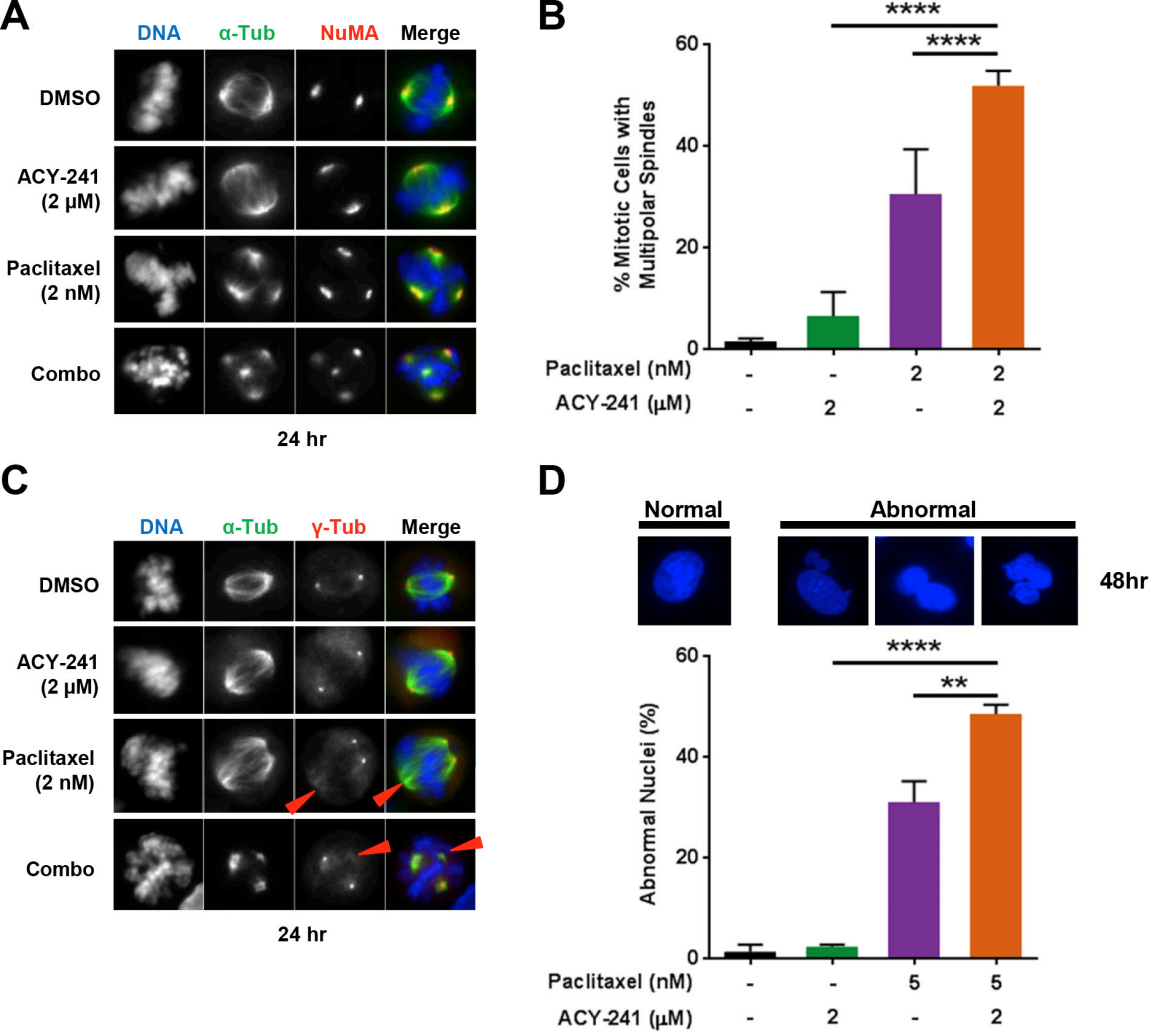
Combination treatment increased the frequency of multipolar mitotic spindle formation and abnormal nuclei TOV-21G cells were treated with vehicle or ACY-241 +/− paclitaxel for 24 hours prior to fixation. (**A**) Staining for α-tubulin (green), NuMA (red), and DNA (blue) demonstrated that all multipolar spindles formed with NuMA-containing spindle poles. (**B**) The frequency of mitotic cells with multipolar spindles was scored in at least 50 cells from each treatment condition. Shown is the mean ± SD of three independent experiments. (**C**) Staining for α-tubulin (green), γ-tubulin (red), and DNA (blue) demonstrated that additional spindle poles formed in the absence of centrosomal γ-tubulin (red arrowheads). (**D**) TOV-21G cells were treated with vehicle, ACY-241, paclitaxel, or the combination for 48 hours prior to fixation. Microtubules and DNA were visualized by staining with anti-α-tubulin antibody (green) and DAPI (blue), respectively. The frequency of interphase cells with visually abnormal nuclei, including satellite nuclei and multinucleation, was scored in at least 100 cells per treatment condition. Shown is the mean ± SD of three independent experiments.

In addition to cell cycle arrest and apoptosis, treatment with low concentrations of paclitaxel was previously shown to result in formation of multipolar mitotic spindles during mitosis, subsequently resulting in aberrant cell divisions and cell death [8, 9]. Consistent with this previous observation, α-tubulin staining demonstrated that the frequency of multipolar mitotic spindle formation in paclitaxel (2 nM) treated TOV-21G ovarian cancer cells increased to 15.2% (*n* = 30/196 mitotic cells), compared with 3.1% (*n* = 2/65) in control treated cells (Supplementary Figure S3). The frequency of multipolar spindle formation in either ACY-241 (2 μM) or ACY-1215 (2 μM) treated cells also increased to 10.7% (*n* = 6/56) and 12.6% (*n* = 11/87), respectively. Importantly, a greater than additive increase in the frequency of multipolar spindle formation to 38.6% (*n* = 61/158) and 41.3% (*n* = 150) was observed in response to combination treatment with either ACY-241 or ACY-1215, respectively (Supplementary Figure S3). To better assess multipolar spindle formation, co-staining was performed for the spindle pole marker Nuclear Mitotic Apparatus (NuMA) [9, 30] and α-tubulin to visualize individual spindle poles in mitotic TOV-21G cells. This immunostaining demonstrated that all observed multipolar α-tubulin spindles formed with NuMA-containing spindle poles (Figure 6A) and confirmed that combination treatment with ACY-241 significantly increased the frequency of multipolar spindle formation from 30.4% with single agent paclitaxel treatment to 51.5% with combination treatment (*p* = 0.0047; Figure 6B). Interestingly, other microtubule stabilizing drugs, including taccalonolides and sagopilone (fully synthetic epothilone B), also induce multipolar spindle formation in a dose-dependent manner [10, 31]. Consistent with this, treatment of TOV-21G cells with epothilone (0.5 nM) increased the frequency of multipolar mitotic spindle formation to 29.9% (*n* = 38/127 mitotic cells) relative to 0.9% (1/107) in control treated cells (Supplementary Figure S4). Combination treatment with epothilone and ACY-241 resulted in a greater than additive increase in the frequency of multipolar spindle formation to 51.3% (*n* = 59/115), suggesting that ACY-241 may be efficacious in combination with a broad spectrum of microtubule stabilizing drugs (Supplementary Figure S4).

Multipolar spindles are commonly associated with abnormal centrosome number [32], thus co-staining was performed for α-tubulin and the centrosome marker γ-tubulin to further characterize multipolar spindle formation in TOV-21G cells. Double staining showed that multipolar spindles formed upon paclitaxel or combination treatment did not always co-localize with γ-tubulin (Figure 6C), suggesting that multipolar spindle formation occurs independent of centrosome amplification.

### Multipolar cell divisions are associated with cell death and nuclear abnormalities

Multipolar spindle formation frequently results in multipolar cell division, which can be followed by cell death [33]. To directly test whether cell death occurs after multipolar spindle formation upon treatment with ACY-241 and paclitaxel, live cells were observed by time-lapse microscopy for 48 hours following treatment. Consistent with enhanced multipolar spindle formation by ACY-241 (Figure 6A, 6B), live cell imaging also showed that combination treatment increased the frequency of multipolar cell division compared to each single agent (Supplementary Figure S5A). As specific examples, Supplementary Figure S5B and Supplementary Video S1 track two representative mitotic cells through mitosis over 48 hours of combination treatment. The first cell clearly proceeded through multipolar cell division (Supplementary Figure S5Ba and S5Ba’, inside yellow circle) to produce two daughter cells, one of which was binucleated due to immediate cell fusion. Chromosomal fragmentation (Supplementary Figure S5Bb and S5Bc, dashed yellow circle) and cell morphology (Supplementary Figure S5Bb’ and S5Bc’) show that these two daughter cells died at 8 and 12 hours after entering anaphase, respectively (see also Video S1). A second cell also proceeded through multipolar cell division (Supplementary Figure S5B, red circle) to generate daughter cells that then fused into one large, multinucleated cell that eventually died 17 hours after entering anaphase (Supplementary Figure S5Be and S5Be’, dashed red circle). A third cell death also occurred in the observation area (Supplementary Figure S5Bd and S5Bd’, dashed green circle) of a binucleated cell that moved into the observation area (Supplementary Figure S5B, green circle), suggesting it may be a daughter cell generated from an additional aberrant mitosis. These live cell imaging data are consistent with combination treatment resulting in multipolar cell division that can lead directly to atypical cells and cell death.

While multipolar cell division can be followed by cell death, a subset of cells with multipolar spindles can still complete mitosis and generate G1 daughter cells with abnormal nuclei [33]. Consistent with the increased frequency of multipolar spindle formation, combination treatment with ACY-241 and paclitaxel also significantly increased the frequency of cells with visually abnormal nuclei, including satellite nuclei and multinucleation (Figure 6D). This finding of more frequent aberrant mitoses is consistent with the observed increase in cell cycle arrest, aberrant DNA content, and increased cell death, likely contributing to the increased anti-cancer efficacy of this combination.

## DISCUSSION

Paclitaxel is a widely used standard of care agent for the treatment of solid tumors, though incomplete clinical responses are commonly seen. There is thus a need for enhanced therapeutic activity in the form of novel drugs that are well tolerated in combination with paclitaxel. ACY-241 is a novel orally available selective HDAC6 inhibitor under clinical development in combination with pomalidomide and dexamethasone in multiple myeloma (NCT 02400242). In this study, ACY-241 treatment in combination with paclitaxel increased the frequency of multipolar spindle formation during mitosis, which was associated with aberrant mitosis and altered DNA content. Combination treatment with ACY-241 further suppressed cancer cell proliferation and increased cell death relative to either single agent, and significantly suppressed tumor growth in solid tumor xenograft models.

Paclitaxel is commonly accepted to enhance microtubule polymerization and reduce microtubule dynamic instability [12, 16]. In the absence of dynamic instability microtubules are no longer able to correctly attach to kinetochores, resulting in unattached kinetochores which then activate the spindle assembly checkpoint (SAC), a signal transduction cascade that arrests mitotic progression by inhibiting the anaphase-promoting complex/cyclosome [34]. However, recent studies demonstrate that while high doses of paclitaxel induce mitotic arrest, clinically relevant low concentrations of paclitaxel increased the frequency of multipolar spindle formation and subsequently increased the rate of aberrant mitosis and cell death [9]. In fact, paclitaxel has been shown to increase the frequency of multipolar spindle formation in xenograft tumors [35].

Formation of bipolar mitotic spindles is required for correct segregation of daughter chromosomes during mitosis. However, multipolar spindles are commonly observed in human tumor specimens, frequently in association with abnormal centrosome number [32]. However, multipolar spindle formation induced by paclitaxel does not occur via these routes as tumors from patients treated with paclitaxel show multipolar spindles in the absence of centrosome amplification [9]. In this prior study, NuMA staining demonstrated the presence of additional spindles, but these additional spindles did not always colocalize to centrosomes containing γ-tubulin [9]. Likewise, in the present study double staining of α-tubulin with NuMA or γ-tubulin confirmed that all spindles in paclitaxel-induced multipolar spindles contained NuMA but did not all contain γ-tubulin, indicating that enhanced multipolar spindle formation due to combination drug treatment occurred independent of centrosome amplification. Future studies exploring the mechanism by which paclitaxel induces formation of multipolar mitotic spindles will potentially elucidate additional mechanisms of this drug combination.

HDAC inhibitors impact a wide range of biological functions through modulation of multiple signaling pathways, including regulating expression of genes critical for cell cycle progression, remodeling chromatin by increasing acetylation of histones, and directly acetylating proteins that control cell cycle progression [18]. In some cases, high concentrations of HDAC inhibitors induced hyperacetylation of histones and triggered G2/M arrest [36, 37]. The dose level of ACY-241 used here did not significantly increase M phase arrest either as a single agent or in combination with paclitaxel (Supplementary Figures S2, S5 and Supplementary Video S1) despite demonstrating pharmacological inhibition of both HDAC6 and Class I HDACs (Figure 1B). Several studies previously demonstrated that inhibition of HDACs effectively disrupts SAC function and caused mitotic delay of 2–3 hours, but did not induce stable mitotic arrest (> 10 hour) in combination with either taxol or nocodazole treatment [38–41]. This observation is in line with the results described here (Supplementary Figures S2 and S5), where combination treatment with ACY-241 did reduce cell cycle progression but without evidence of prolonged M phase accumulation. These findings suggest that the observed aberrant mitotic figures may eventually bypass the SAC and progress through mitosis, as was observed via live-cell time-lapse imaging, thus resulting in cell death or daughter cells with abnormal DNA content and/or nuclear morphology.

There are multiple potential mechanisms by which inhibition of various HDAC isozymes could cooperate with paclitaxel to disrupt mitotic spindle formation. Similar to prior studies with non-selective pan-HDAC inhibitors that inhibit HDAC6 as well as other HDACs, ACY-241 in combination with paclitaxel significantly increases acetylation of α-tubulin [25, 29]. Hyperacetylation of α-tubulin due to HDAC6 inhibition has been shown to reduce microtubule dynamic instability [13], suggesting the ACY-241/paclitaxel combination may act to further suppress microtubule dynamic instability. Indeed, it was shown that the non-selective pan-HDAC inhibitor Trichostatin A has cooperative effects with paclitaxel on both apoptosis and microtubule instability in papillary serous endometrial cancer cells [42]. Beyond potentially impacting microtubule stability, HDAC6 inhibition could also impact mitotic spindle formation due to its known effects on the microtubule motor protein dynein, where the HDAC6/dynein complex regulates the transport of ubiquitinated proteins along microtubules towards aggresomes [43]. Interestingly, dynein also plays an important role during spindle assembly and spindle pole formation as depletion of dynein led to unfocused microtubules at spindle poles and poor centrosomal attachment, resulting in multipolar spindle formation [44]. Beyond HDAC6, several previous studies showed that inhibition of Class I HDACs could weaken SAC function through increasing acetylation of histones followed by dis-localization of chromosomal passenger complex (CPC) proteins, potentially impairing microtubule attachment to the centromere [39, 40, 45]. SAC activation can suppress multipolar mitoses by providing additional time for appropriate formation of bipolar mitotic spindles [46]. Thus, it is possible that HDAC inhibitors may weaken SAC activation therefore reducing the role of the SAC in suppressing spindle multipolarity, resulting in indirect enhancement of multipolar spindle formation. Future studies could explore the contribution(s) of individual HDAC isozymes to the observed combination efficacy with paclitaxel.

Both HDAC inhibition and paclitaxel treatment impact diverse cellular pathways in both interphase and mitosis to interrupt the growth and proliferation of cancer cells. The findings presented here identify an enhancement of abnormal cell division from combination treatment with paclitaxel and the selective HDAC inhibitor ACY-241, though additional mechanisms likely also contribute to the observed anti-cancer activity in cellular and animal models of various solid tumor types. For example, though not tested in this study, combination treatment may also mechanistically interact to directly reduce progression from G1 to S phase. Together, these findings supported the initiation of an ongoing Phase 1b clinical trial (NCT02551185) to evaluate the safety and preliminary efficacy of ACY-241 in combination with paclitaxel in patients with advanced solid tumors.

## MATERIALS AND METHODS

### Cell Lines and reagents

The ovarian cancer cell line A2780 was obtained from Sigma (Santa Louis, MO), while the ovarian cancer cell line TOV-21G, breast cancer cell lines MDA-MB-231 and T47D, and pancreatic carcinoma cell line MiaPaCa-2 were obtained from ATCC (Manassas, VA). A2780 and MDA-MB-231 cells were cultured in RPMI1640 media supplemented with 10% fetal bovine serum (FBS), TOV-21G cells were cultured in RPMI1640 media with 15% FBS, T47D cells were cultured in Dulbecco's Modified Eagle's Medium (DMEM) supplemented with 10% FBS, and MiaPaCa-2 cells were cultured in DMEM with 10% FBS and 2.5% horse serum (HS).

Ricolinostat and ACY-241 were synthesized by ChemPartner (Shanghai, China). Paclitaxel and epothilone B were obtained from Selleck Chemicals (Houston, TX). All agents were dissolved in DMSO for *in vitro* use.

### HDAC enzymatic assays

ACY-241 was dissolved and subsequently diluted in assay buffer [50 mM HEPES, pH 7.4, 100 mM KCl, 0.001% Tween-20, 0.05% BSA, and 20 μM tris(2-carboxyethyl)phosphine] to 6-fold the final concentration. HDAC enzymes were diluted to 1.5-fold of the final concentration in assay buffer and pre-incubated with ACY-241 for 10 min before the addition of the substrate. The amount of FTS (HDAC1, HDAC2, HDAC3, and HDAC6) or MAZ-1675 (HDAC4, HDAC5, HDAC7, HDAC8, and HDAC9) used for each enzyme was equal to the Michaelis constant (Km), as determined by a titration curve. FTS or MAZ-1675 was diluted in assay buffer to 6-fold the final concentration with 0.3 M sequencing grade trypsin (Sigma). The substrate/trypsin mix was added to the enzyme/compound mix and the plate was shaken for 60 sec and then placed into a SpectraMax M5 microtiter plate reader. The enzymatic reaction was monitored for release of 7-amino-4-methoxy-coumarin over 30 min, after deacetylation of the lysine side chain in the peptide substrate, and the linear rate of the reaction was calculated.

### Animal studies

All animal studies were performed in female athymic nude mice (Crl:NU(NCr)-Foxn1^nu^) housed in appropriate animal care facilities during the experimental period at an Association for Assessment and Accreditation of Laboratory Animal Care International (AAALAC) certified facility at Charles River Discovery Services (Morrisville, NC). All procedures complied with the recommendations of the Guide for Care and Use of Laboratory Animals. Xenografts were initiated by subcutaneously injecting 100 μL of cell suspensions, equivalent to 1 × 10^7^ cells for TOV-21G tumor cell lines, into the right flank of 7-week-old mice. Tumors for all mice were allowed to grow until a volume of 100 to 150 mm^3^ before randomization (cohort sizes *n* ≥ 7) and treatment initiation. Ricolinostat and ACY-241 were dosed via intraperitoneal injection at 50 mg/kg once daily for five days, followed by two days off, for three consecutive weeks (50 mg/kg IP QD 5/2 × 3wk), while paclitaxel was dosed via intraperitoneal injection at 10 mg/kg once daily for five consecutive days (10 mg/kg IP QD × 5) or intravenously at 10 mg/kg every other day for a total of five doses (10 mg/kg IV QOD × 5). Tumor volumes and body weights were measured twice weekly throughout the duration of the experiment and tumor growth inhibition (TGI) was assessed at the end of the third cycle of therapy. Differences in tumor volume are indicated by *p* values obtained by performing a one-way ANOVA at the final tumor measurement time point, followed by a Tukey multiple hypothesis correction.

### Cell proliferation assays

The growth inhibitory effect of single agent ACY-241 or paclitaxel was assessed by measuring 3-(4,5-dimethylthiazol-2-yl)-5-(3-carboxymethoxyphenyl )-2-(4-sulfophenyl)-2H-tetrazolium (MTS; CellTiter 96^®^ AQueous One Solution; Promega; Madison, WI, USA) dye absorbance. Cells (A2780, TOV-21G, MDA-MB-231) from 72 hour cultures were pulsed with 5 μL of CellTiter 96^®^ AQueous One Solution in each well. The 384-well plates were incubated at 37°C for 5 hours, and absorbance was read in triplicate at a wavelength of 490 nm (with background correction using readings at 650 nm) on a spectrophotometer (Molecular Devices Corp.; Sunnyvale, CA, USA). The mean and standard deviation were then calculated.

The inhibitory effect of the drug combination on cell proliferation was assessed by crystal violet staining. Briefly, cells were seeded in replicate 12-well plates at 5,000 (MiaPaCa-2 and TOV-21G) or 20,000 (T47D) cells per well, allowed to attach overnight, and then treated with the indicated concentrations of ACY-241 and/or paclitaxel for up to 9 days. On each of days 0, 5, 7, and 9, the culture medium was removed from one plate, the cells were washed with Phosphate Buffered Saline (PBS), fixed with 4% w/v paraformaldehyde at room temperature for 10 min, and then stained with 0.05% w/v crystal violet (Sigma) for 15 min. The cells were then washed with water, after which the water was removed and the plates were dried at room temperature. After collection of samples from all time points, the crystal violet was extracted in 10% v/v acetic acid, and absorbance was read in triplicate at a wavelength of 540 nm on a spectrophotometer (Molecular Devices Corp.; Sunnyvale, CA, USA). The mean and standard deviation were then calculated.

### IncuCyte^®^ proliferation and apoptosis assay

Proliferation and Caspase 3/7 activation apoptosis assays were performed at Essen Bioscience (Ann Arbor, MI, USA). NucLight Red expressing MDA-MB-231 cells were plated at 2,500 cells/well in 96-well plates for 24 hours prior to treating with DMSO, ACY-241, and/or paclitaxel. Caspase 3/7 reagent was added (time = 0) and cells were imaged every 2 hours for 96 hours in an IncuCyte^®^ Zoom Live Cell Analysis system at 37°C in 5% CO_2_. Proliferation was assessed through absolute count of NucLight Red labeled cells over time while activation of apoptosis was assessed through total Caspase 3/7 positive objects over time. Each condition was performed in triplicate. Kinetic trace graphs of NucLight Red Proliferation and Caspase 3/7 apoptosis were generated by plotting the mean with SEM at each time point in Prism (Graphpad, La Jolla, CA, USA).

### Cell cycle assessment

Cells cultured in the presence of ACY-241 and/or paclitaxel were incubated for 60 min in the presence of 10 μM 5-ethynyl-2’-deoxyuridine (EdU) at 37°C. After EdU incorporation, cells were washed and resuspended in fixative solution using the Click-iT^®^ EdU Alexa Fluor^®^ 488 Flow Cytometry Assay Kit (Life Technologies, Grand Island, NY). Cellular DNA was stained using FxCycle™ Far Red Stain (Life Technologies) and cells analyzed on an Attune NxT Flow cytometer (ThermoFisher Scientific) or Cytomics FC 500 MPL flow cytometer (Beckman Coulter; Indianapolis, IN). Flow cytometry data were analyzed using FlowJo software (TreeStar; Ashland, OR). Dual-parameter plots were generated for Alexa Fluor^®^ 488–labeled EdU fluorescence (indicating newly synthesized DNA) and FxCycle™ Far Red Stain fluorescence (indicating relative DNA content). The generated plots have a typical inverted U-shaped pattern that identifies proliferating cells with bright EdU staining and nonproliferating cells with dim EdU staining that are either in G1 phase (with 2N DNA content) or in G2/M phase (with 4N DNA content). Cell death is indicated by the subG1 population, which are the nonproliferating cells with dim EdU staining and low DNA content (< 2N; corresponding to the lower left corner of the dual-parameter plot).

### Immunoblotting

Cells cultured in the presence of ACY-241 and/or paclitaxel were washed and lysed using radio-immunoprecipitation assay (RIPA) lysis buffer containing 5 mM NaF, 2 mM Na_3_VO_4_, 1 mM PMSF and complete protease inhibitor cocktail (Thermo Scientific). Lysates were sonicated 2 to 3 times for 30 sec in medium (Diagenode, Bioruptor 300).

For western blot analysis, equal amounts of protein were loaded and size separated on Bolt™ 4 to 12% Bis-Tris Plus Gels. Proteins were subsequently transferred onto polyvinylidene difluoride (PVDF) membrane using an iBlot^®^ 2 Dry Blotting System (Invitrogen). Immunoblotting was carried out according to standard protocols with antibodies against histone H3 acetyl-lysine 56 (abcam), histone-H4, pantropic (Millipore), acetyl-α-tubulin (Sigma); and β-Actin (Sigma). Antigen-antibody complexes were detected using secondary antibodies conjugated with horseradish peroxide (HRP) and visualized using enhanced chemiluminescence (GE Healthcare; Piscataway, NJ).

For WES analysis, lysates were separated by an automated capillary-based electrophoresis system (WES, ProteinSimple, San Jose, CA). All procedures were performed according to the manufacturer's recommendations using the supplied reagents. Briefly, after determination of protein concentration of each lysate, 0.4 mg of total protein (4 μL) was mixed with 1 μL of 5X fluorescent master mix and heated at 95°C for 5 min. The samples, blocking reagent, wash buffer, primary antibodies, secondary antibodies, and chemiluminescent substrate were dispensed into designated wells in the manufacturer provided microplate. Following plate loading the separation and immunodetection was performed automatically using default settings. The resulting data were analyzed using Compass software (ProteinSimple).

### Immunofluorescence

Cells grown on glass coverslips were fixed in 4% paraformaldehyde for 15 min, followed by permeabilization with 0.5% Triton X-100 in PBS for 5 min. The cells were then incubated with α-tubulin (DM1A) mouse monoclonal antibody (Cell Signaling Technology, Danvers, MA), γ-tubulin (GTU-88) mouse monoclonal antibody (Sigma-Aldrich, St. Louis, MO) or NuMA (GT3611) mouse monoclonal antibody (Thermo Scientific, Rockford, IL) followed by Alexa Fluor 488 or 546 conjugated secondary antibodies (Invitrogen, Carlsbad, CA). Images were obtained with a Zeiss AX10 microscope (Carl Zeiss Micro-Imaging, Inc., Jena, Germany) equipped with a 20 X, 40X, or 63 X objective and standard filter set.

### Live cell imaging

Time-lapse imaging was obtained on a Zeiss AX10 live-cell microscope coupled with a CO_2_ and temperature-maintenance system, and a time-lapse recording system supporting multi-location time series acquisition (SlideBook6 x64, Intelligent Imaging Innovations, Inc., Ringsby, CT). After staining with 10 nM Hoechst33342, TOV-21G cells were incubated in 10% CO_2_ at 37°C and time-lapse images were acquired at 2 min intervals up to 48 hours with a CoolSNAP HQ2 CCD camera (Photometrics, Tucson, AZ).

### Statistical analysis

All *in vitro* experiments were performed in triplicate and repeated at least 3 times unless indicated otherwise. The statistical significance of differences was determined using a one-way ANOVA test with post-hoc Tukey multiple comparison correction. All statistical analyses were performed using Prism v6.4 software (GraphPad).

## SUPPLEMENTARY MATERIALS FIGURES AND VIDEO





## References

[1] Jordan MA, Wilson L (2004). Microtubules as a target for anticancer drugs. Nat Rev Cancer.

[2] Rowinsky EK, Donehower RC (1995). Paclitaxel (taxol). N Engl J Med.

[3] Pazdur R, Kudelka AP, Kavanagh JJ, Cohen PR, Raber MN (1993). The taxoids: paclitaxel (Taxol) and docetaxel (Taxotere). Cancer Treat Rev.

[4] Bhalla KN (2003). Microtubule-targeted anticancer agents and apoptosis. Oncogene.

[5] Ibrado AM, Kim CN, Bhalla K (1998). Temporal relationship of CDK1 activation and mitotic arrest to cytosolic accumulation of cytochrome C and caspase-3 activity during Taxol-induced apoptosis of human AML HL-60 cells. Leukemia.

[6] Wang TH, Wang HS, Soong YK (2000). Paclitaxel-induced cell death: where the cell cycle and apoptosis come together. Cancer.

[7] Jordan MA, Ojima I, Rosas F, Distefano M, Wilson L, Scambia G, Ferlini C (2002). Effects of novel taxanes SB-T-1213 and IDN5109 on tubulin polymerization and mitosis. Chem Biol.

[8] Hernandez-Vargas H, Palacios J, Moreno-Bueno G (2007). Molecular profiling of docetaxel cytotoxicity in breast cancer cells: uncoupling of aberrant mitosis and apoptosis. Oncogene.

[9] Zasadil LM, Andersen KA, Yeum D, Rocque GB, Wilke LG, Tevaarwerk AJ, Raines RT, Burkard ME, Weaver BA (2014). Cytotoxicity of paclitaxel in breast cancer is due to chromosome missegregation on multipolar spindles. Sci Transl Med.

[10] Risinger AL, Riffle SM, Lopus M, Jordan MA, Wilson L, Mooberry SL (2014). The taccalonolides and paclitaxel cause distinct effects on microtubule dynamics and aster formation. Mol Cancer.

[11] Gardner MK, Zanic M, Howard J (2013). Microtubule catastrophe and rescue. Curr Opin Cell Biol.

[12] Mitchison T, Kirschner M (1984). Dynamic instability of microtubule growth. Nature.

[13] Asthana J, Kapoor S, Mohan R, Panda D (2013). Inhibition of HDAC6 deacetylase activity increases its binding with microtubules and suppresses microtubule dynamic instability in MCF-7 cells. J Biol Chem.

[14] Song Y, Brady ST (2015). Post-translational modifications of tubulin: pathways to functional diversity of microtubules. Trends Cell Biol.

[15] Jordan MA, Kamath K (2007). How do microtubule-targeted drugs work? An overview. Curr Cancer Drug Targets.

[16] Yvon AM, Wadsworth P, Jordan MA (1999). Taxol suppresses dynamics of individual microtubules in living human tumor cells. Mol Biol Cell.

[17] Hubbert C, Guardiola A, Shao R, Kawaguchi Y, Ito A, Nixon A, Yoshida M, Wang XF, Yao TP (2002). HDAC6 is a microtubule-associated deacetylase. Nature.

[18] Gabrielli B, Brown M (2012). Histone deacetylase inhibitors disrupt the mitotic spindle assembly checkpoint by targeting histone and nonhistone proteins. Adv Cancer Res.

[19] Harada T, Hideshima T, Anderson KC (2016.). Histone deacetylase inhibitors in multiple myeloma: from bench to bedside. Int J Hematol.

[20] Lee JH, Marks PA (2010). Histone deacetylase inhibitors in the therapy of cancer: much to learn. Epigenomics.

[21] West AC, Johnstone RW (2014). New and emerging HDAC inhibitors for cancer treatment. J Clin Invest.

[22] Bradner JE, West N, Grachan ML, Greenberg EF, Haggarty SJ, Warnow T, Mazitschek R (2010). Chemical phylogenetics of histone deacetylases. Nat Chem Biol.

[23] Dizon DS, Damstrup L, Finkler NJ, Lassen U, Celano P, Glasspool R, Crowley E, Lichenstein HS, Knoblach P, Penson RT (2012). Phase II activity of belinostat (PXD-101), carboplatin, and paclitaxel in women with previously treated ovarian cancer. Int J Gynecol Cancer.

[24] Ramalingam SS, Maitland ML, Frankel P, Argiris AE, Koczywas M, Gitlitz B, Thomas S, Espinoza-Delgado I, Vokes EE, Gandara DR, Belani CP (2010). Carboplatin and Paclitaxel in combination with either vorinostat or placebo for first-line therapy of advanced non-small-cell lung cancer. J Clin Oncol.

[25] Catalano MG, Poli R, Pugliese M, Fortunati N, Boccuzzi G (2007). Valproic acid enhances tubulin acetylation and apoptotic activity of paclitaxel on anaplastic thyroid cancer cell lines. Endocr Relat Cancer.

[26] Liu Z, Tong Y, Liu Y, Liu H, Li C, Zhao Y, Zhang Y (2014). Effects of suberoylanilide hydroxamic acid (SAHA) combined with paclitaxel (PTX) on paclitaxel-resistant ovarian cancer cells and insights into the underlying mechanisms. Cancer Cell Int.

[27] Robertson FM, Chu K, Boley KM, Ye Z, Liu H, Wright MC, Moraes R, Zhang X, Green TL, Barsky SH, Heise C, Cristofanilli M (2013). The class I HDAC inhibitor Romidepsin targets inflammatory breast cancer tumor emboli and synergizes with paclitaxel to inhibit metastasis. J Exp Ther Oncol.

[28] Yee AJ, Bensinger WI, Supko JG, Voorhees PM, Berdeja JG, Richardson PG, Libby EN, Wallace EE, Birrer NE, Burke JN, Tamang DL, Yang M, Jones SS (2016). Ricolinostat plus lenalidomide, and dexamethasone in relapsed or refractory multiple myeloma: a multicentre phase 1b trial. Lancet Oncol.

[29] Zuco V, M De Cesare, Cincinelli R, Nannei R, Pisano C, Zaffaroni N, Zunino F (2011). Synergistic antitumor effects of novel HDAC inhibitors and paclitaxel in vitro and in vivo. PLoS One.

[30] Radulescu AE, Cleveland DW (2010). NuMA after 30 years: the matrix revisited. Trends Cell Biol.

[31] Eschenbrenner J, Winsel S, Hammer S, Sommer A, Mittelstaedt K, Drosch M, Klar U, Sachse C, Hannus M, Seidel M, Weiss B, Merz C, Siemeister G (2011). Evaluation of activity and combination strategies with the microtubule-targeting drug sagopilone in breast cancer cell lines. Front Oncol.

[32] Maiato H, Logarinho E (2014). Mitotic spindle multipolarity without centrosome amplification. Nat Cell Biol.

[33] Pihan GA (2013). Centrosome dysfunction contributes to chromosome instability, chromoanagenesis, and genome reprograming in cancer. Front Oncol.

[34] Foley EA, Kapoor TM (2013). Microtubule attachment and spindle assembly checkpoint signalling at the kinetochore. Nat Rev Mol Cell Biol.

[35] Orth JD, Kohler RH, Foijer F, Sorger PK, Weissleder R, Mitchison TJ (2011). Analysis of mitosis and antimitotic drug responses in tumors by in vivo microscopy and single-cell pharmacodynamics. Cancer Res.

[36] Bolden JE, Peart MJ, Johnstone RW (2006). Anticancer activities of histone deacetylase inhibitors. Nat Rev Drug Discov.

[37] Qiu L, Burgess A, Fairlie DP, Leonard H, Parsons PG, Gabrielli BG (2000). Histone deacetylase inhibitors trigger a G2 checkpoint in normal cells that is defective in tumor cells. Mol Biol Cell.

[38] Dowling M, Voong KR, Kim M, Keutmann MK, Harris E, Kao GD (2005). Mitotic spindle checkpoint inactivation by trichostatin a defines a mechanism for increasing cancer cell killing by microtubule-disrupting agents. Cancer Biol Ther.

[39] Magnaghi-Jaulin L, Eot-Houllier G, Fulcrand G, Jaulin C (2007). Histone deacetylase inhibitors induce premature sister chromatid separation and override the mitotic spindle assembly checkpoint. Cancer Res.

[40] Stevens FE, Beamish H, Warrener R, Gabrielli B (2008). Histone deacetylase inhibitors induce mitotic slippage. Oncogene.

[41] Warrener R, Beamish H, Burgess A, Waterhouse NJ, Giles N, Fairlie D, Gabrielli B (2003). Tumor cell-selective cytotoxicity by targeting cell cycle checkpoints. FASEB J.

[42] Dowdy SC, Jiang S, Zhou XC, Hou X, Jin F, Podratz KC, Jiang SW (2006). Histone deacetylase inhibitors and paclitaxel cause synergistic effects on apoptosis and microtubule stabilization in papillary serous endometrial cancer cells. Mol Cancer Ther.

[43] Kawaguchi Y, Kovacs JJ, McLaurin A, Vance JM, Ito A, Yao TP (2003). The deacetylase HDAC6 regulates aggresome formation and cell viability in response to misfolded protein stress. Cell.

[44] Morales-Mulia S, Scholey JM (2005). Spindle pole organization in Drosophila S2 cells by dynein, abnormal spindle protein (Asp), and KLP10A. Mol Biol Cell.

[45] Ma Y, Cai S, Lu Q, Lu X, Jiang Q, Zhou J, Zhang C (2008). Inhibition of protein deacetylation by trichostatin A impairs microtubule-kinetochore attachment. Cell Mol Life Sci.

[46] Kwon M, Godinho SA, Chandhok NS, Ganem NJ, Azioune A, Thery M, Pellman D (2008). Mechanisms to suppress multipolar divisions in cancer cells with extra centrosomes. Genes Dev.

