# Supplementary material for: Selective HDAC inhibition by ACY-241 enhances the activity of paclitaxel in solid tumor models
Source: Oncotarget. 2016 Dec 1;8(2):2694–707. doi: 10.18632/oncotarget.13738 (PMC5356834; doi:10.18632/oncotarget.13738)
Supplement: Supplementary file 1 [file oncotarget-08-2694-s001.pdf]

Selective HDAC inhibition by ACY-241 enhances the activity of paclitaxel in solid tumor models

Supplementary Materials

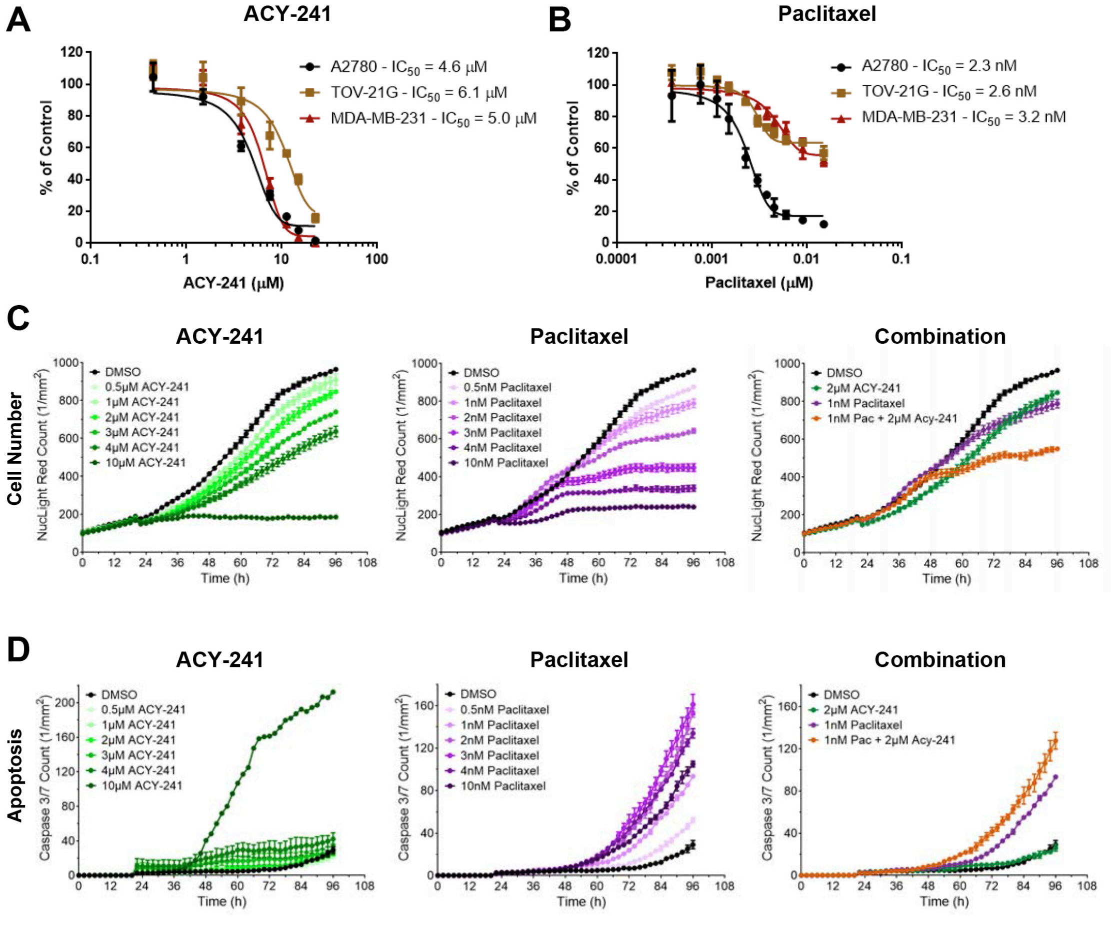

Supplementary Figure S1: Effect of ACY-241 and Paclitaxel on cell viability and induction of apoptosis in solid tumor cell lines. Cell viability was assessed by an MTS-based assay in the A2780, TOV-21G, and MDA-MB-231 solid tumor cancer cell lines by treating cells in the presence of ACY-241 (**A**) or paclitaxel (**B**) for 3 days. The IC_{50} for each agent in each cell line is indicated in the legend of each graph. Real-time kinetic trace graphs of (**C**) cell proliferation and (**D**) induction of apoptosis were generated in the MDA-MB-231 breast cancer cell line. Proliferation was assessed through absolute nuclear count over time, and apoptosis was assessed through total Caspase 3/7 positive objects over 96 hours of treatment with the indicated doses of ACY-241 (left panels), paclitaxel (middle panels), or the combination (right panels). The mean and SEM of three replicate samples are shown.

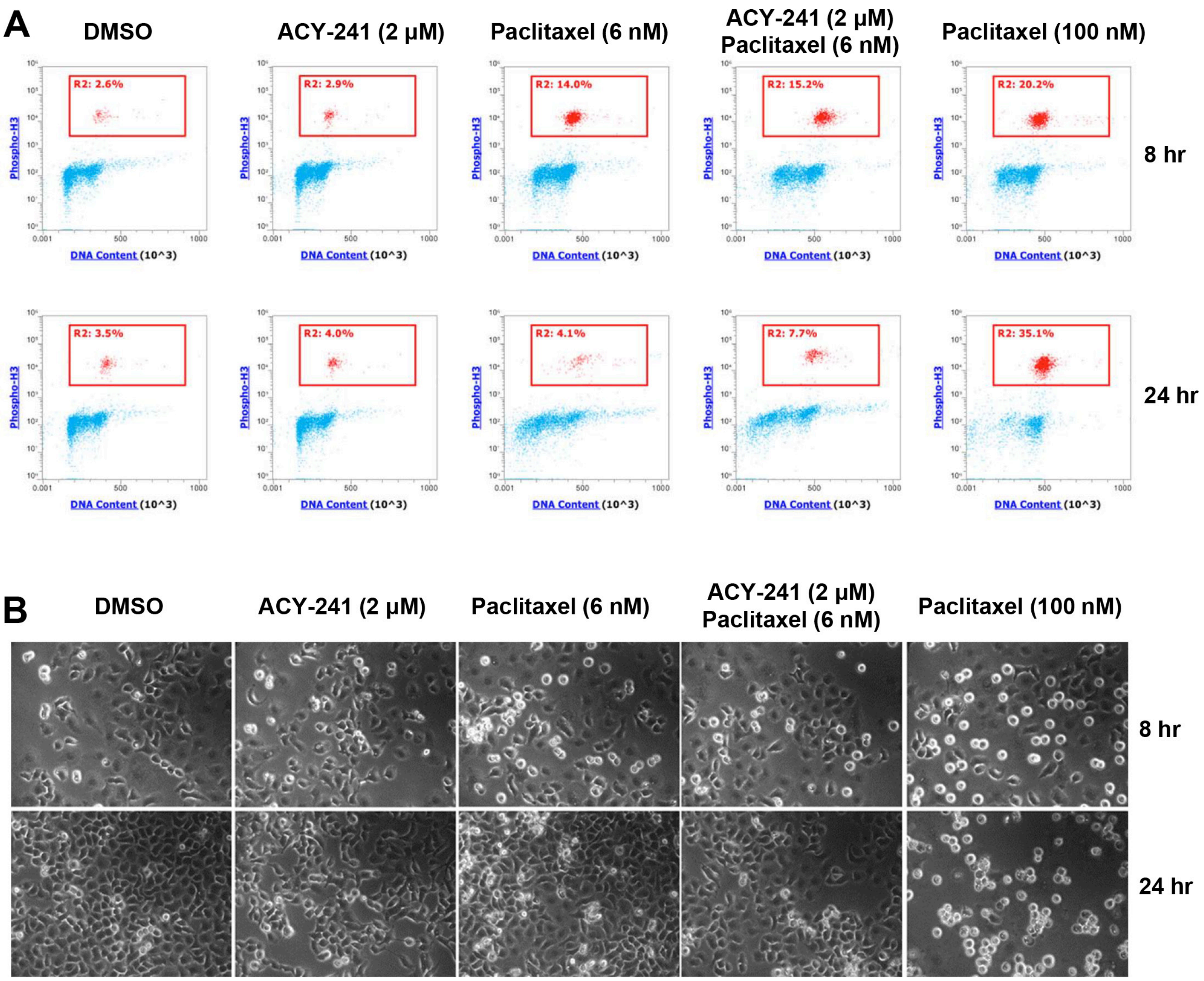

Supplementary Figure S2: Combination treatment with ACY-241 and paclitaxel did not induce sustained mitotic arrest. (**A**) Dual parameter phospho-histone H3 (y axis, M phase cells) and FxCycle Far Red (x axis, DNA content) staining indicate the percentage of cells in M phase (red box) after treatment with low dose of paclitaxel (6 nM) and/or ACY-241 (2 μM) for the indicated times. High dose treatment of paclitaxel (100 nM) served as a positive control for M phase arrest. (**B**) Phase contrast micrographs were taken at the indicated time points after treatment of the samples in panel (A).

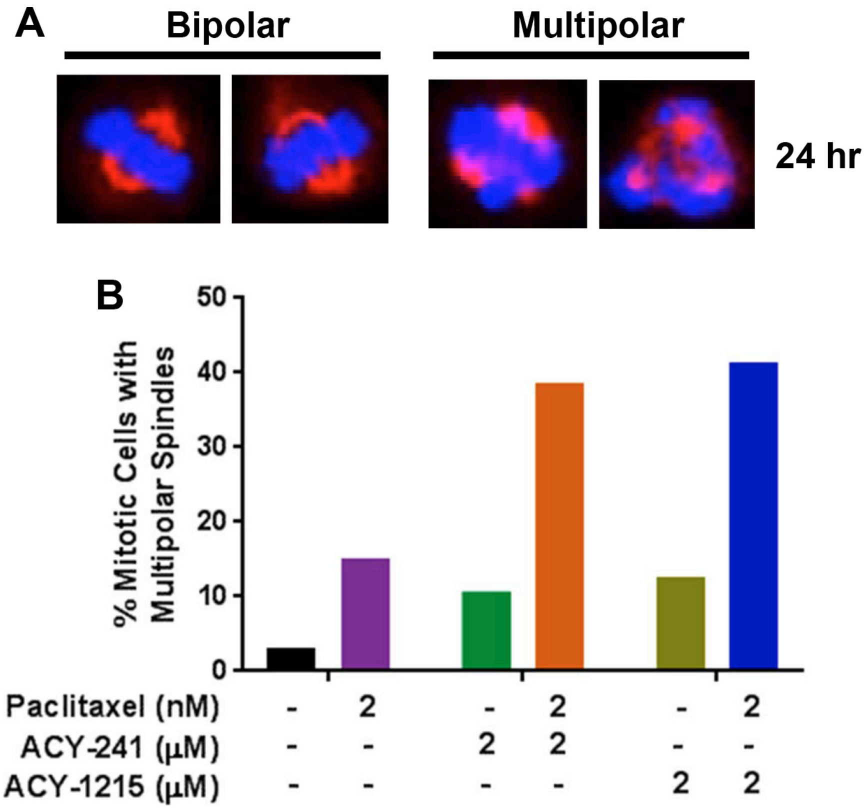

Supplementary Figure S3: Combination treatment increased the frequency of multipolar mitotic spindle formation. TOV-21G cells were treated with vehicle, ACY-241, ACY-1215, paclitaxel, or each combination for 24 hours prior to fixation. (**A**) Staining for α-tubulin (red) and DNA (blue) identified mitotic cells with multipolar spindles. (**B**) The frequency of multipolar spindle formation in M phase cells was scored in at least 50 cells per treatment condition.

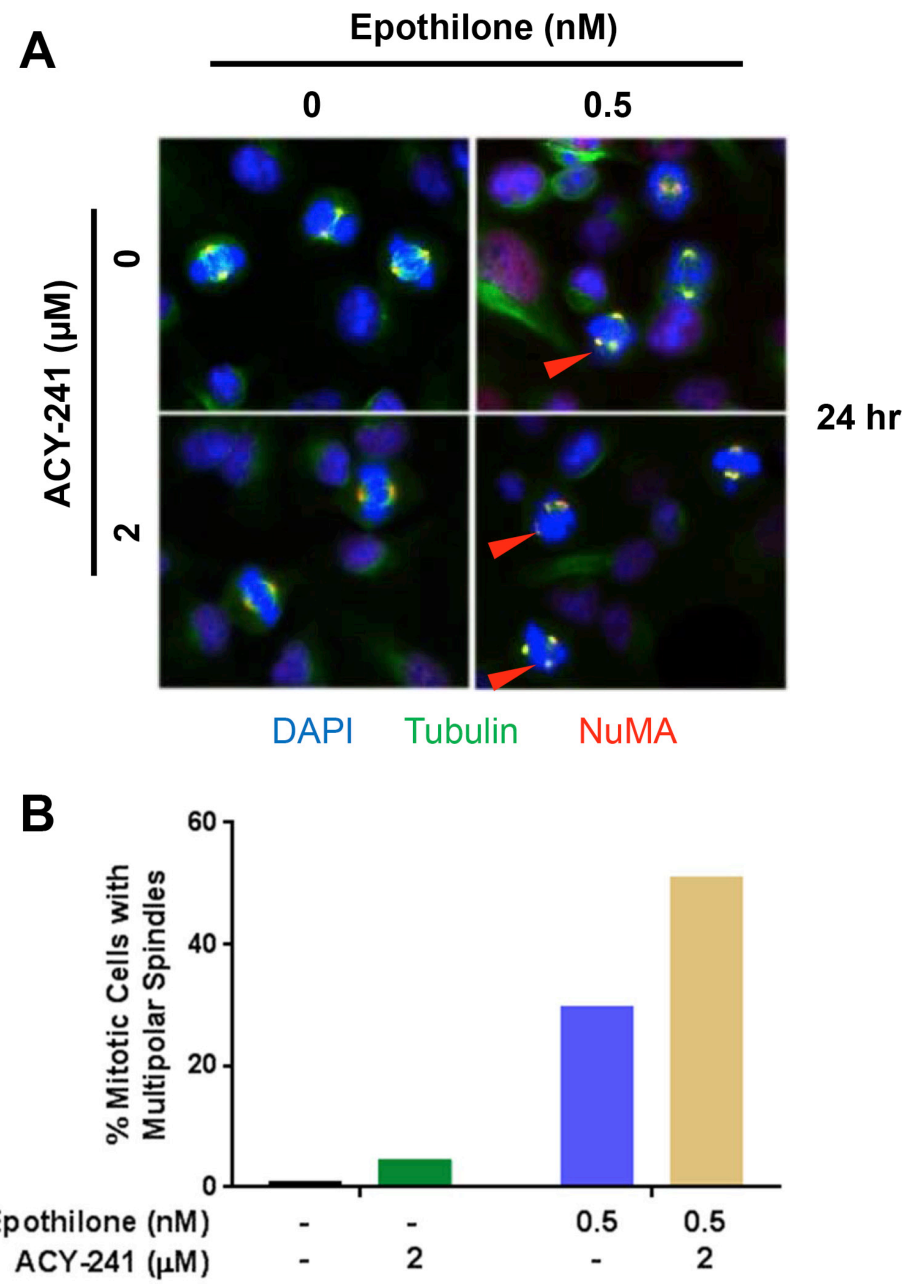

Supplementary Figure S4: ACY-241 enhances multipolar spindle formation in combination with epothilone. TOV-21G cells were treated with vehicle or ACY-241 +/− epothilone for 24 hours prior to fixation. (**A**) Immunostaining for α-tubulin (green), NuMA (red), and DNA (blue). (**B**) The frequency of mitotic cells with multipolar spindles was scored in at least 100 cells from each treatment condition.

A

	Mitotic Cells	Unresolved Mitosis	Bipolar Division	Multipolar Division
Control	23	0/23	23/23	0/23 (0%)
ACY-241 (2 µM)	27	0/27	24/27	3/27 (12.5%)
Paclitaxel (5 nM)	31	5/31	17/26	9/26 (34.6%)
Combo	13	1/13	0/12	12/12 (100%)

B

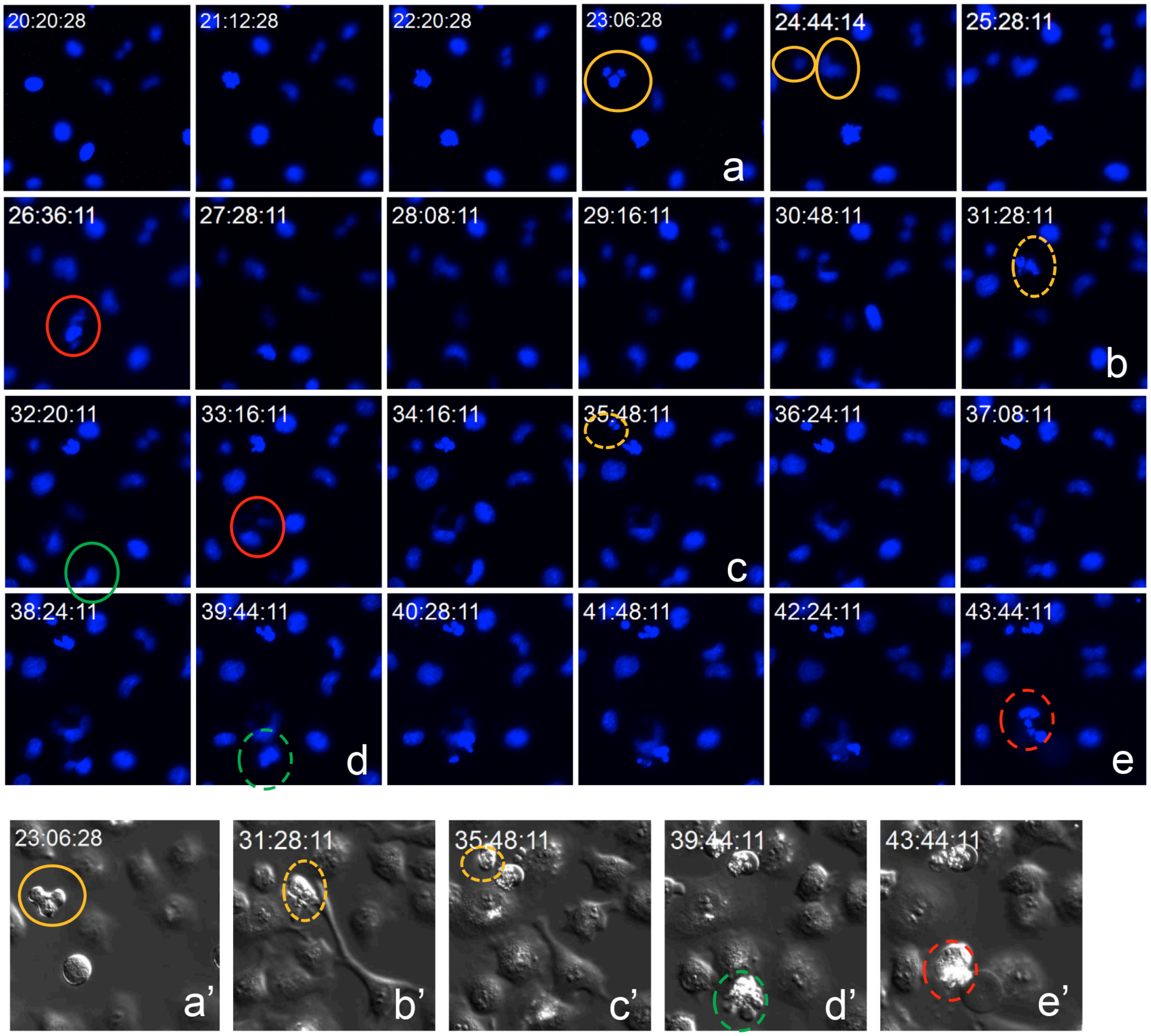

Supplementary Figure S5: Multipolar cell division is observed upon combination treatment with ACY-241 and paclitaxel. TOV-21G cells were treated with vehicle, ACY-241 (2 µM), paclitaxel (5 nM), or the combination for up to 48 hours and monitored by live cell time-lapse microscopy. (**A**) The number of observed mitotic cells, unresolved mitoses, and frequency of bipolar and multipolar division from resolved mitosis were assessed. (**B**) Fluorescent and matched phase-contrast time-lapse images of representative mitoses in TOV-21G cells treated with the drug combination. Numbers show elapsed time after drug treatment (hour:minute:second), the color of circles differentiates individual cells being monitored, and dashed circles indicate observation of cell death. Panels labeled with lower case letters (a-e) identify timepoints with matched phase contrast images below (a'-e').

Supplementary Video S1: Cell death is observed to follow multipolar cell division after combination treatment with ACY-241 and paclitaxel. TOV-21G cells were treated with ACY-241 (2 μM) plus paclitaxel (5 nM) and fluorescent and matched phase-contrast time-lapse images were acquired at 2 minute intervals over a 48 hour period and compiled into a movie at 20 frames per second. Numbers show elapsed time after drug treatment (hour:minute:second), and representative frames are presented in Supplementary Figure S5B. See Supplementary_Video_S1
